# Preharvest application of ethephon and postharvest UV-B radiation improve quality traits of beetroot (*Beta vulgaris* L. ssp. *vulgaris*) as source of colourant

**DOI:** 10.1186/s12870-018-1556-2

**Published:** 2018-12-03

**Authors:** Gregorio Barba-Espin, Stephan Glied-Olsen, Tsaneta Dzhanfezova, Bjarne Joernsgaard, Henrik Lütken, Renate Müller

**Affiliations:** 1 Centro de Edafología y Biología Aplicada del Segura, CSIC, Grupo de Biotecnología de Frutales, Departamento de Mejora Vegetal, P.O. Box 164, E-30100 Murcia, Spain; 2 Section for Crop Sciences, Department of Plant and Environmental Sciences, Faculty of Science, University of Copenhagen, Hoejbakkegaard Alle 9-13, 2630 Taastrup, Denmark; 3 Natural Colors Division, Chr. Hansen A/S, Agern Allé 24, 2970 Hørsholm, Denmark

**Keywords:** Beetroot, Betalain biosynthetic pathway, Betanin, Ethephon, UV-B radiation, Vulgaxanthin

## Abstract

**Background:**

Betanins have become excellent replacers for artificial red-purple food colourants. Red beet (*Beta vulgaris* L. spp. *vulgaris*) known as beetroot, is a rich source of betalains, which major forms are betanin (red to purple) and vulgaxanthin (yellow). Betalains and phenolic compounds are secondary metabolites, accumulation of which is often triggered by elicitors during plant stress responses. In the present study, pre-harvest applications of ethephon (an ethylene-releasing compound) and postharvest UV-B radiation were tested as elicitors of betalains and phenolic compounds in two beetroot cultivars. Their effects on quality parameters were investigated, and the expression of biosynthetic betalain genes in response to ethephon was determined.

**Results:**

Ethephon was applied as foliar spray during the growth of beetroot, resulting in increased betanin (22.5%) and decreased soluble solids contents (9.4%), without detrimental effects on beetroot yield. The most rapid accumulation rate for betanin and soluble solids was observed between 3 and 6 weeks after sowing in both untreated and ethephon-treated beetroots. Overall, the expression of the betalain biosynthetic genes (*CYP76AD1*, *CYP76AD5*, *CYP76AD6* and *DODA1*), determining the formation of both betanin and vulgaxanthin, increased in response to ethephon treatment, as did the expression of the betalain pathway activator *BvMYB1*. In the postharvest environment, the use of short-term UV-B radiation (1.23 kJ m^− 2^) followed by storages for 3 and 7 days at 15 °C resulted in increased betanin to vulgaxanthin ratio (51%) and phenolic content (15%).

**Conclusions:**

The results of this study provide novel strategies to improve key profitability traits in betalain production. High betanin concentration and high betanin to vulgaxanthin ratio increase the commercial value of the colourant product. In addition, lowering soluble solids levels facilitates higher concentration of beetroot colour during processing. Moreover, we show that enhanced betanin content in ethephon-treated beetroots is linked to increased expression of betalain biosynthetic genes.

## Background

Over the past 20 years, the market of natural food colours has grown substantially owing to legal restrictions and consumer concerns [1, 2]. Currently, the market for natural colours accounts for more than 55% of the total food colour market. Some of the most common natural pigments are carotenoids, chlorophylls, anthocyanins and betalains. Betalains are nitrogen containing pigments which substitute anthocyanins in plants within the Caryophyllales order [3]. The only source of betalain approved for use as food colourant in the U.S. and European Union are the roots of red beet (*Beta vulgaris* L. ssp. *vulgaris*), known as beetroot. Nowadays, beetroot colourants are widely used in dairy products, frozen desserts and meat [4].

Beetroot colourants are commercialised as either juice concentrate (produced by vacuum-concentration of juice to 60–65% total solids) or dehydrated powder. In addition to the lower stability of natural pigments, the main constrains to extraction and use of beetroot concentrates as food colourants are the relatively low concentration of betalain in root juice and the high content of sugars. Since sugar contents are 80 to 200 times higher than betalain contents in the root, lowering soluble solids levels in the red beet would facilitate concentration of beetroot colour during processing, increasing the commercial value of the product [4, 5]. Compared to anthocyanins, betalains have higher water solubility and tinctorial strength [6]. Additionally, beetroot colour is brighter and more stable over the pH range 4–7 [7], although on the other hand it displays lower heat stability.

Betalains comprise two groups of water-soluble pigments: the red–purple betacyanidins and the yellow betaxanthins. Betacyanidins are conjugates of cyclo-DOPA and betalamic acid, and betaxanthins are conjugates of amines or amino acids and betalamic acid. Betacyanidins are normally glycosylated, in which case they are called betacyanins. In mature beetroots, red–purple betacyanins comprise the major part of pigments, and of these a single compound, betanin, comprises 75–95%. Yellow betaxanthins account for a minor part of beetroot pigments, vulgaxanthin I being the most abundant form [8, 9]. Betacyanins are more stable than betaxanthins, both at room temperature [10] and upon heating [11].

Betalains account for 70–100% of the total phenolic content of beetroot [12]. Other phenolic compounds in red beet include gallic, syringic, caffeic acids and others [13]. Phenolic compounds provide strong free radical-scavenging properties to beetroot, acting as natural antioxidants in the prevention of diseases associated with oxidative stress [9, 14]. Moreover, betalains and total phenolic compounds increase the antioxidant activity of beetroot extracts synergistically [15].

Betalains and phenolic compounds are secondary metabolites, and their accumulation can be affected by abiotic factors or stressors from the environment. To our knowledge, there are no studies reporting increased betalain content by the use of elicitors in red beet plants in vivo. Recently, the betalain biosynthetic pathway of beetroot has been fully elucidated [16, 17]. However, the mechanism by which this pathway is regulated in response to stress remains unknown.

In the present study, ethephon and UV-B radiation were used in pre- and postharvest environments, respectively, as enhancers of betalain content. Ethephon, an ethylene-generating compound, was applied as foliar spray during the growth of red beet. In this respect, preharvest application of ethephon has been reported previously to increase pigmentation in orange and black carrots [18, 19]. In the postharvest environment, the role of UV-B radiation as inductor of phenolic pigments [20, 21] was tested. The effects of ethephon and UV-B radiation were investigated on betalain and total phenolic contents, and on several quality parameters. The expression patterns of the betalain biosynthetic genes were studied in response to ethephon. Along with the practical significance of enhanced betalain content for colour production, this study provides new insights into the regulation of betalain biosynthesis in beetroot.

## Material and methods

### Plant material

Red beet ‘Monty Rz’ and ‘Belushi Rz’ were selected based on their high betanin content from a previous screening on 16 weeks-old beetroots of 15 commercial varieties (Joernsgaard 2015, personal communication). Seeds were provided by Rijk Zwaan (De Lier, Netherlands), and the two cultivars were used in both field and postharvest experiments.

### Field conditions and ethephon treatment

Field trials were conducted at the University of Copenhagen, Hoejbakkegaard (Denmark) in 2015, in accordance with local legislation and international guidelines. Three-row plots were arranged in randomised block designs with three replicates. Small plots (4.5 m-long rows) were harvested a single time, whereas large plots (12 m-long rows) were harvested multiple times form distant row segments. Foliar applications of ethephon (CERONE® brand ETHEPHON, Bayer Crop Science, Leverkusen, Germany) at a concentration of 360 g ha^− 1^ active ingredient were performed as described previously [19]. Ethephon application began 5 weeks after sowing and continued every 3 weeks, with a total of four applications. Standard techniques recommended in red beet crop production were conducted. The sowing dates, ethephon applications and harvest dates of the different trials are specified in Table 1. For further analyses, biological replicates consisted of 20 whole beetroots harvested per plot.

**Table 1:** Sowing dates, harvest dates and ethephon applications of the different field trials conducted during the 2015 growing season in Denmark

Harvest	Trial sowing date	Ethephon treatment dates	Harvest date(s)
Single	16 June 22.06.15	22 July; 12 Aug.; 02 Sept.; 23 Nov.	06 Oct.
	22 June	30 July.; 20 Aug.; 09 Sept.; 30 Sept.	13 Oct.
Multiple	16 June	22 July; 12 Aug.; 02 Sept.; 23 Nov.	13 July; 03 Aug.; 24 Aug.; 14 Sept.; 05 Oct.; 26 Oct.

### Postharvest conditions and UV-B radiation treatment

Beetroots of ‘Monty Rz’ and ‘Belushi Rz’ not subjected to previous field treatment were harvested on 22 May 2015, topped, and stored at 4 °C to be treated the following day. At time zero, beetroots were placed at a distance of 60 cm from the UV-B lamps (Philips Broadband TL40W/12 RS, Eindhoven, Netherlands) and irradiated with a UV-B radiation fluence of 1.23 kJ m^− 2^, corresponding to a UV-B radiation fluence rate of 17.5 W m^− 2^ for 70 s. The whole root surface was exposed by turning the beetroot at the middle of the treatment (35 s). UV-B radiation was measured using a RM-12 Ultraviolet Light Meter equipped with a UVB sensor (Opsytec Dr. Gröbel GmbH, Ettlingen, Germany). After the UV-B radiation treatment, beetroots were stored at 15 °C and 98–100% relative humidity (RH) in darkness, for 3 and 7 days. Each treatment included five biological replicates each consisting of eight beetroots.

### Sample preparation

At harvest, biological replicates consisting of twenty beetroots (field experiment) or eight beetroots (postharvest experiment) were washed and cut in halves. Of these, one pool of halves were homogenised in a 3% sulfuric acid solution (1/1, *w*/w) and subsequently mixed with milliQ water or 70% ethanol as described [19], for the analysis of betalains or total phenolic content, respectively. The complementary beetroot halves were ground to a powder under liquid nitrogen before storage at − 80 °C for further gene expression analyses.

### Determination of betanin (Bn) and vulgaxanthin I (Vx)

Bn and Vx were measured spectrophotometrically as described previously [22] with slight modifications. The beetroot extract was diluted to a proper concentration in 33 mM KH_2_PO_4_ (pH 6.5), and the absorption was measured at 476 nm and 538 nm for Bn and Vx, respectively, by means of a UV-visible spectrophotometer (Thermo Scientific Evolution™ 220, Waltham, MA, USA). Bn and Vx concentrations were expressed in mg kg^− 1^ of root fresh weight (FW), using the corresponding absorbance, molecular weight and extinction coefficient for Bn and Vx.

### Determination of total phenolic content (TPC)

TPC was calculated according to the Folin–Ciocalteau method [23]. Briefly, 100 μL of beetroot extract were mixed with 0.5 mL of Folin–Ciocalteau reagent and 1 mL of 20% (*w*/*v*) sodium carbonate. The samples were thereafter incubated for 2 h in the dark, and the absorbance of the mix was determined at 760 nm using a UV-visible spectrophotometer (Thermo Scientific Evolution™ 220). Based on the measured absorbance, the TPC mg of gallic acid equivalent (GAE) per kg of fresh weight was deduced from the calibration curve.

### Determination of dry matter (DM) and total soluble solids content (TSS)

One mL of beetroot extract was filtered through 0.45 μm membrane filters, and TSS was subsequently measured with a manual refractometer in the 0 to 85% Brix range (Refracto 30PX/GS Mettler-Toledo Inc., OH, USA) operating.

DM was determined after samples were dried to a constant weight at 100 °C for 24 h, based on the difference in mass between the fresh and dry samples. DM was then expressed as a percentage of the dry matter.

### RNA isolation, cDNA synthesis and real-time quantitative PCR

Total RNA was extracted from ground beetroots with RNeasy® Plant Mini Kit (Qiagen, Hilden, Germany) and then treated with DNase I Amplification Grade (Sigma–Aldrich, MO, USA) according to the manufacturers’ instructions, to eliminate residual DNA. Agarose gel electrophoresis and a NanoDrop™ 1000 Spectrophotometer (Thermo Fisher Scientific, MA, USA) were used to evaluated RNA quality and integrity. Two micrograms of RNA from each sample were utilised to synthesise cDNA in a 20 μl reaction volume using the cDNA iScript™ Synthesis Kit (Bio-Rad, Hercules, CA, USA) according to the manufacturer’s instructions.

To assess the expression levels of genes involved in betalain biosynthesis in response to ethephon, primers specific for *Actin*, *CYP76AD1*, *CYP76AD5*, *CYP76AD6*, *DODA1*, and *MYB1* were designed using Primer3 online software and assessed prior to use. The RT-qPCR reactions were conducted as described previously [19], and the relative quantification was performed according to the 2^(−ΔΔCt) method [24]. Primer efficiency was ¨tested by plotting the threshold cycles (Ct) at each concentration against the logarithm of the fold-dilution of the sample. The threshold cycles (Ct) for the target genes were standardised to the *BvActin* Ct (ΔCt) [17]. Nucleotide sequences of primer pairs specific for each gene are provided in Table 2.

**Table 2:** Annotation, accession number and nucleotide sequences of primers to genes used for Real Time q-PCR

Gene annotation	GenBank ID	Forward primer 5′-3′	Reverse primer 5′-3′	Fragment length
*BvActin*	HQ656028.1	ttgctgaccgtatgagcaag	ttctgtggacgattgatgga	192
*BvCYP76AD1*	HQ656023	ttcacggccctttaatatcg	tggcaagcatcaagtctttg	250
*BvCYP76AD5*	KM592961.1	gcgcatagacaatccaaggt	gaatggggaagaaatcagca	241
*BvCYP76AD6*	KT962274	gctaaccgaaccattcctga	tatcgacgggttgcattttt	223
*BvDODA1*	HQ656027	ggaaccagaattggcaagaa	gagccaatgctcgtcctaag	209
*BvMYB1*	JF432080.1	atcgtcggcaaccataaaag	atgcccacaagttcacaaca	248

Primers were designed using Primer3 online software

### Statistical analyses

At least three biological replicates were utilised, and data were subjected to statistical analysis using the R 3.0.0 statistical package (MA, USA). Data from the accumulation curves were analysed with the lmer function of the lme4 R package (MA, USA). Treatments were compared using one- or two-way analysis of variance (ANOVA) followed by a Tukey post-hoc test. When the assumption of a normal distribution of the data was rejected, data were analysed using the Kruskal-Wallis test followed by a Nemenyi post-hoc test. *p* ≤ 0.05 was considered to indicate statistical significance.

## Results

### Effect of ethephon field-treatment on betalain pigments, TPC and yield data

**Fig. 1:**
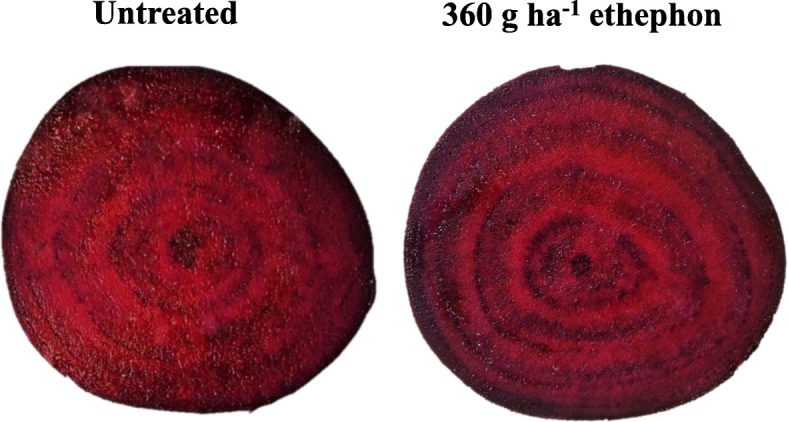
Cross sections of roots of untreated and 360 g ha^− 1^ ethephon-treated red beet plants at 16-weeks after sowing

In the first part of the present study, the effect of ethylene as a preharvest elicitor of betalain pigments was investigated in beetroots foliar-sprayed with ethephon. First, the Bn and Vx content was initially analysed in the roots of 16 week-old plants. Overall, ethephon-treated plants exhibited increased Bn content in both cultivars studied. In contrast, Vx content did not vary significantly between untreated and treated plants (Table 3). In general, similar pigment concentrations were obtained in both experiment repetitions. Transversal root sections did not display visual differences between untreated and treated red beets (Fig. 1). The mean root Bn content in treated plants of ‘Monty Rz’ ranged from 2166 ± 72 to 2458 ± 33 mg kg^− 1^ FW, representing an increase of 25% compared with the values of untreated plants (1872 ± 105 to 1972 ± 83 mg kg^− 1^ FW). Similarly, Bn content in roots of treated plants of ‘Belushi Rz’ displayed mean values of 1575 ± 26 to 1584 ± 21 mg kg^− 1^ FW, whereas the corresponding values in untreated plants ranged from 1240 ± 22 to 1418 ± 27 mg kg^− 1^ FW (Table 3), which represents an increase of 20% in average. As a result of increased Bn and unchanged Vx concentrations, the Bn:Vx increased substantially in both cultivars upon ethephon treatment (Table 3). Ethephon treated beetroots of ‘Monty Rz’ displayed mean Bn:Vx of 6.3, representing 34% increase compared with the ratio of untreated plants (4.7), whereas the corresponding ratio in ‘Belushi Rz’ (6.1) represented 36% increase compared with the ratio of untreated plants (4.5). Relative to the TPC, both red beet cultivars displayed increases of 22.5% in average for the two experiment repetitions. Roots from ‘Monty Rz’ red beets showed the highest TPC concentration following ethephon treatment (Table 3). Ethephon applications did not alter significantly DM for both cultivars (Table 3). The opposite occurred with the mean TSS, which displayed lower values in treated plants of ‘Monty Rz’ (11.4%) and ‘Belushi Rz’ (12.9%), compared with the values of untreated roots. Ethephon applications did not vary beetroot yield in tonnes per hectare (data not shown).

**Table 3:** Betanin (Bn) and vulgaxanthin (Vx) contents, betanin to vulgaxanthin ratio (Bn:Bx), total phenolic content (TPC) and yield data in roots of ethephon-treated beetroot plants in trials harvested at a single time-point (16 weeks after sowing)

Trial sowing date	Cultivar	Ethephon (g ha^− 1^)	Betalains (mg kg^− 1^ FW)			TPC	TSS	DM
			Bn	Vx	Bn:Vx			
16/06/15	‘Monty Rz’	0	1872 ± 105^b^	432 ± 32^a^	4.38	1385 ± 43^b^	16.13 ± 0.06^a^	12.37 ± 0.07^a^
		360	2166 ± 72^a^	386 ± 57^a^	5.79	1643 ± 43^a^	14.25 ± 0.17^b^	11.63 ± 0.23^a^
	‘Belushi Rz’	0	1240 ± 49^b^	339 ± 22^a^	3.67	960 ± 33^b^	15.41 ± 0.17^a^	11.34 ± 0.38^a^
		360	1575 ± 57 ^a^	274 ± 26^a^	5.82	1243 ± 25^a^	13.86 ± 0.06^b^	10.58 ± 0.10^a^
22/06/15	‘Monty Rz’	0	1972 ± 83^b^	405 ± 43^a^	4.94	1588 ± 32^b^	18.07 ± 0.01^a^	13.97 ± 0.20^a^
		360	2458 ± 33^a^	364 ± 37^a^	6.88	1980 ± 11^a^	16.41 ± 0.19^b^	13.07 ± 0.42^a^
	‘Belushi Rz’	0	1418 ± 50^b^	276 ± 27^a^	5.28	1159 ± 35^b^	17.57 ± 0.10^a^	13.07 ± 0.40^a^
		360	1584 ± 24^a^	247 ± 21^a^	6.42	1349 ± 65^a^	17.07 ± 0.15^b^	12.14 ± 0.13^a^

*TSS* total soluble solids content, *DM* dry matter. Data represent the mean ± SE, *n* = 3. Different letters indicate statistical significance according to Tukey’s test (*p* ≤ 0.05);

### Betalain and TPC accumulation during beetroot growth

**Fig. 2:**
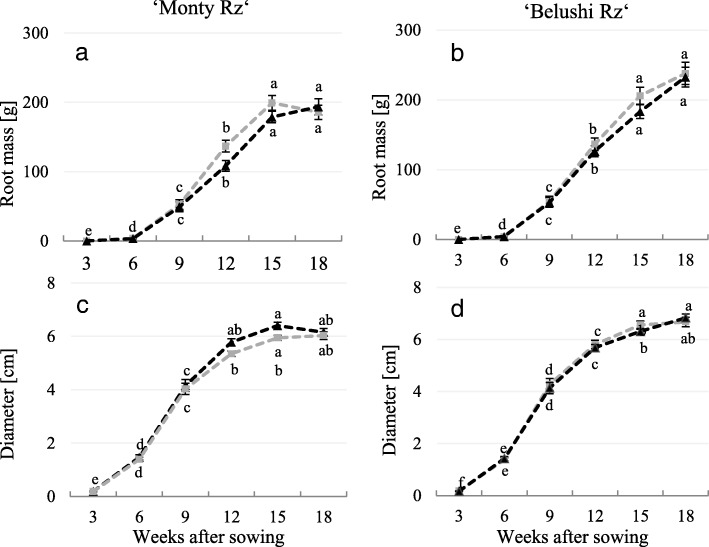
**a**, **b** Root weight and **c**, **d** transversal diameter monitored in untreated and 360 g ha^− 1^ ethephon-treated red beet plants (3–18 weeks after sowing). Different letters indicate statistical significance according to Tukey’s test (*p* ≤ 0.05). Data represent the mean ± SE, *n* = 3

Bn and Vx content, TPC, TSS, DM and root size were monitored in response to 360 g ha^− 1^ ethephon, from 13 July to 26 October 2015 (3, 6, 9, 12, 15 and 18 weeks after sowing) (Fig. 2). There were no significant differences in root mass (Fig. 2a and b) and diameter (Fig. 2c and d) between untreated and treated red beets of both cultivars at each harvest point. Roots of ‘Belushi Rz’ reached higher values of root mass (237 ± 14 g) than those for ‘Monty Rz’ (199 ± 11 g).

**Fig. 3:**
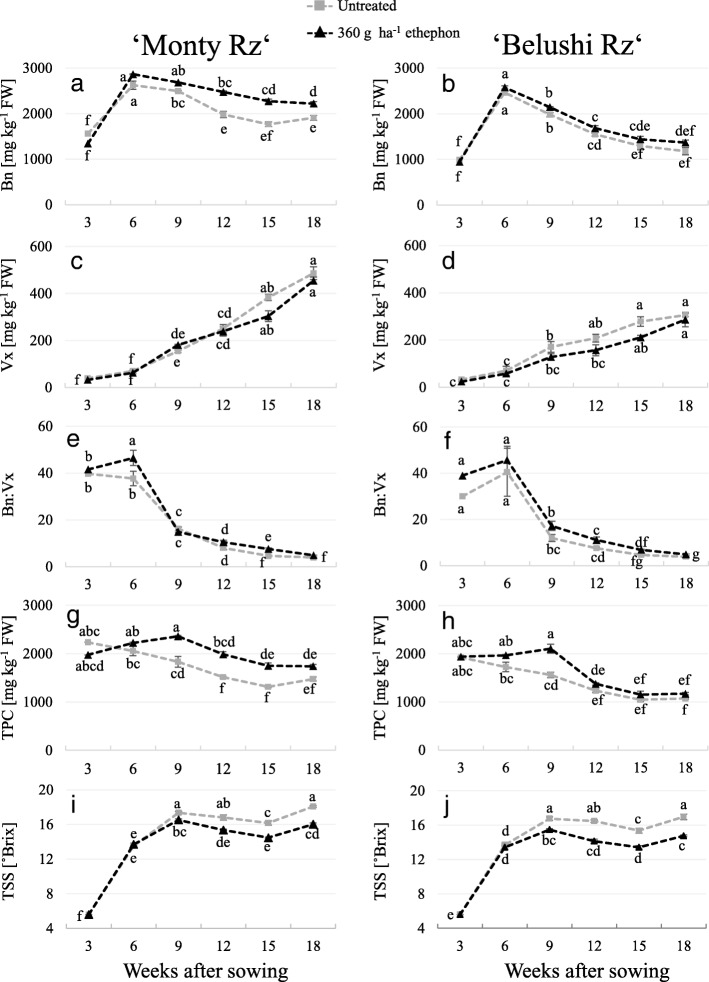
**a**, **b** Betanin (Bn) content, **c**, **d** vulgaxanthin (Vx) content, **e**, **f** betanin to vulgaxanthin ratio (Bn:Vx), total phenolic content (**g**, **h**), and (**i**, **j**) total soluble solids content (TSS) monitored in roots of untreated and 360 g ha^− 1^ ethephon-treated red beets (3–18 weeks after sowing). Different letters indicate statistical significance according to Tukey’s test (*p* ≤ 0.05). Data represent the mean ± SE, *n* = 3

Overall, Bn content of treated plants was higher at every harvest point (Fig. 3a and b). The opposite occurred with the mean root Vx, which displayed lower values in untreated plants during root growth (Fig. 3c and d). Bn and Vx content followed different kinetics during root growth. Bn content displayed a peak 6 weeks after sowing (2867 ± 11 and 2577 ± 33 mg kg^− 1^ FW in ‘Monty Rz’ and ‘Belushi Rz’, respectively), followed by a gradual decrease until the end of the growing period (Fig. 3a and b). In contrast, Vx content increased over time, reaching 486 ± 27 and 306 ± 6 mg kg^− 1^ FW in ‘Monty Rz’ and ‘Belushi Rz’, respectively, at 18 weeks after sowing (Fig. 3c and d). The highest Bn:Vx in both cultivars (46) was reached at early stages of root growth, 6 weeks after sowing, followed by a drop until the end of the growing period (Fig. 3e and f). Differences in Bn concentration per FW between untreated and treated roots were enhanced when data were expressed per DM (data not shown). In roots of untreated red beet, TPC decreased over time in both cultivars. In contrast, roots of ethephon-treated plants showed enhanced TPC accumulation, displaying a peak 9 weeks after sowing, followed by pronounced decrease (Fig. 3g and h). Based on the levels of betalains and TPC, it can be concluded that non-betalaininc phenolic compounds represent a minor percentage of the total TPC (data not shown).

Overall, TSS displayed lower values in roots of ethephon-treated red beets. The kinetics of TSS in both cultivars, and untreated and elicitor-treated plants, could be divided into three stages: an initial stage with a rapid rate of accumulation (3 to 9 weeks after sowing), a transitional stage determined by a small decrease in TSS, and a later increase during the last 3 weeks of growth, reaching 18.1 and 17.0 °Brix in roots of treated plants of ‘Monty Rz’ and ‘Belushi Rz’, respectively (Fig. 3i and j). In addition, the most rapid accumulation rate for betanin (Fig. 3a and b) and soluble solids (Fig. 3i and j) was observed between 3 and 6 weeks after sowing in both untreated and ethephon-treated beetroots.

### Relative expression of betalain biosynthesis-related genes

**Fig. 4:**
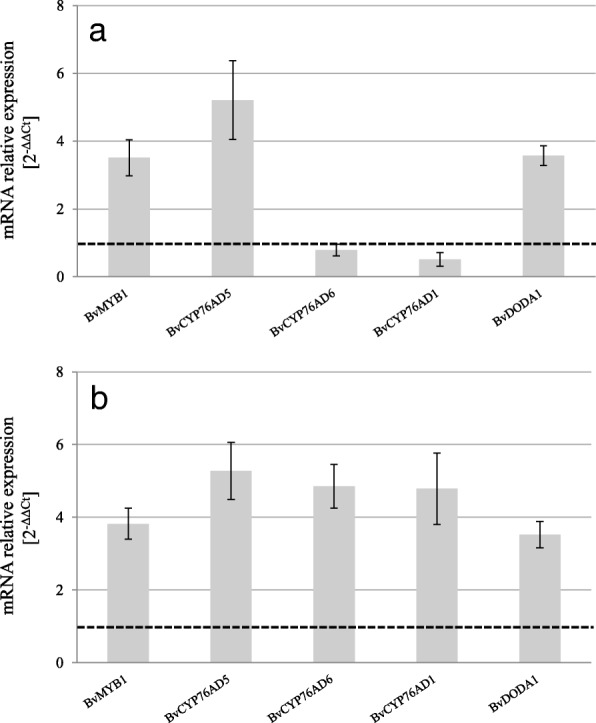
‘Fold changes in target gene expression in roots of 360 g ha^− 1^ ethephon-treated red beet plants of ‘Monty Rz’ (**a**) and ‘Belushi Rz’ (**b**) relative to untreated plants (dashed horizontal line) at 16 weeks after sowing. The relative expression of target genes is determined according to the 2^(−ΔΔCt) method. Threshold cycles (Ct) for target genes are standardised to the *BvActin* Ct (ΔCt). Expression levels of target genes in untreated carrots were assigned an arbitrary value of 1. Data represent mean ± SE, *n* = 3

The expression of the four known betalain biosynthetic genes (*BvDODA1*, *BvCYP76AD1*, *BvCYP76AD5* and *BvCYP76AD6*) and the betalain pathway activator *BvMYB1* was quantified in untreated and ethephon-treated beetroots of ‘Monty Rz’ (Fig. 4a) and ‘Belushi Rz’ (Fig. 4b), at 16 weeks after sowing. The expression of the five genes studied was enhanced in treated roots of ‘Belushi Rz’ (2.6- to 7.9-fold) compared with the levels in untreated plants (Fig. 4a). In treated roots of ‘Monty Rz’ transcripts of *BvMYB1*, *BvDODA1*, *BvCYP76AD5* accumulated to a lesser extent (2.0- to 5.8-fold) compared with the levels in untreated plants, whereas the accumulation of *BvCYP76AD1* and *BvCYP76AD6* was either constitutive or below the levels in untreated plants (Fig. 4b).

### Effect of UV-B postharvest treatment on betalain pigments, TPC and quality treats

**Fig. 5:**
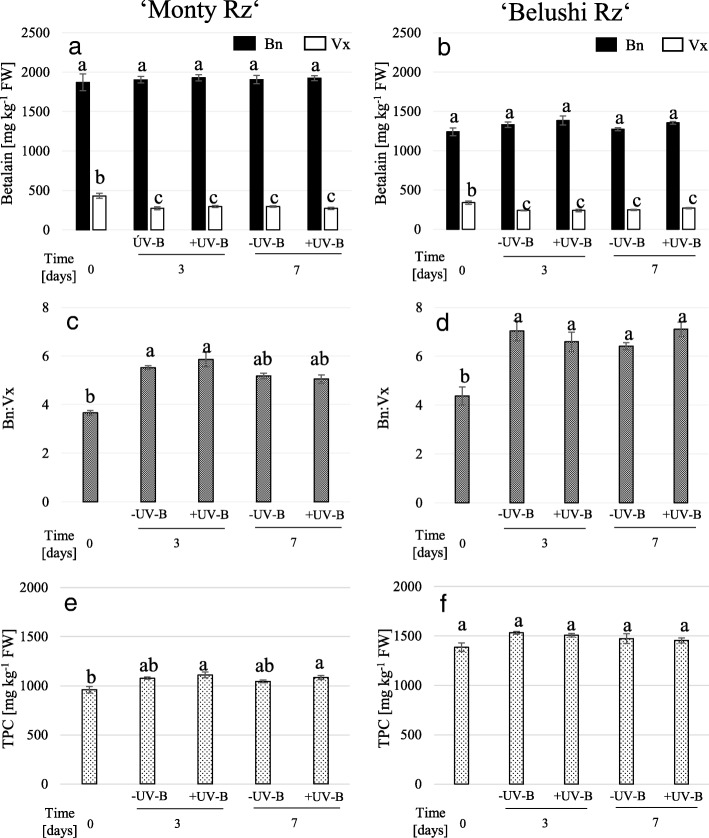
Betalain and total phenolic contents in 16 weeks old beetroots of ‘Monty Rz’ (**a**, **c**, **e**) and ‘Belushi Rz’ (**b**, **d**, **f**) treated with (+UV-B) or without (-UV-B) 1.23 kJ m^− 2^ for 70 s, followed by storage at 15 °C and 98–100% relative humidity in darkness, for 3 and 7 days. Bn: betanin content; Vx: vulgaxanthin content; Bn:Vx: betanin to vulgaxanthin ratio; TPC: total phenolic content. **a**, **b**, **e**, **f**: Different letters denote statistical significance according to Tukey’s test (*p* ≤ 0.05). **c**, **d**: Different letters denote statistical significance according to Nemenyi’s test (*p* ≤ 0.05). Data represent the mean ± SE, *n* = 5

In the second part of the present study, the effect of UV-B radiation as an elicitor of betalain pigments and TPC was investigated in beetroots during short-time postharvest storage. Neither 3 and 7 days of storage nor UV-B radiation treatment changed Bn content of ‘Monty Rz’ and ‘Belushi Rz’ (Fig. 5a and b). In contrast, Vx content decreased significantly under the given storage conditions, both after 3 and 7 days of storage, regardless of whether roots were untreated or subjected to UV-B radiation (Fig. 5a and b). At time zero, Vx content displayed levels of 432 ± 32 mg kg^− 1^ FW in ‘Monty Rz’ and 339 ± 22 mg kg^− 1^ FW in ‘Belushi Rz’, whereas after 7 days of storage, Vx content decreased to 273 ± 14 and 269 ± 7 mg kg^− 1^ FW in ‘Monty Rz’ and ‘Belushi Rz’, respectively. Consequently, Bn:Vx increased in both cultivars, from ratios of 4.38 and 3.67 at time 0 to values of 7.11 and 5.86 at time 7, which represent increases in Bn:Vx of 55 and 47% in ‘Monty Rz’ and ‘Belushi Rz, respectively (Fig. 5 and d). The effect of UV-B radiation on TPC differed between cultivars. In ‘Belushi Rz’, the application of UV-B radiation and the following storage for 7 days induced an increase of 15% of TPC in ‘Belushi Rz’ (Fig. 5f), which indicates that minor phenolic compounds, rather than betalains, increased as a response to the UV-B radiation treatment. In contrast, neither storage time nor UV-B radiation treatment increased TPC significantly in roots of ‘Monty Rz’ (Fig. 5e).

**Fig. 6:**
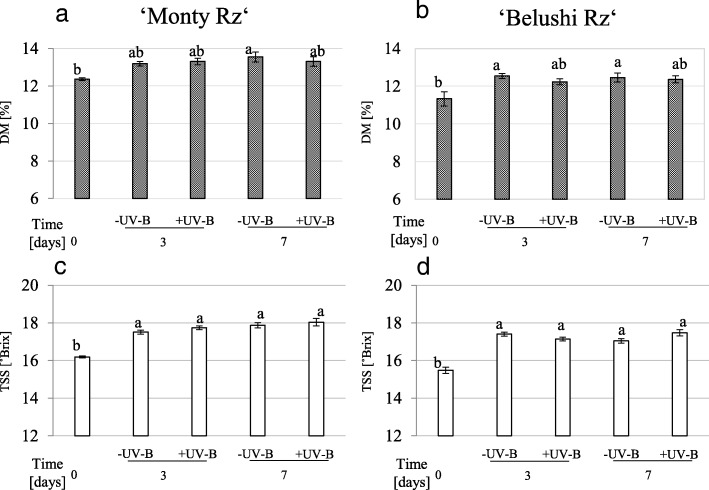
**a**, **b** Dry matter (DM), and (c, d) total soluble solids content (TSS) in 16 weeks old beetroots of ‘Monty Rz’ and ‘Belushi Rz’ treated with (+UV-B) or without (-UV-B) 1.23 kJ m^− 2^ UV-B radiation for 70 s, followed by storage at 15 °C and 98–100% relative humidity in darkness, for 3 and 7 days. Different letters denote statistical significance according to Tukey’s test (*p* ≤ 0.05). Data represent the mean ± SE, *n* = 5

Minimal root weight losses below 1% were recorded upon 7 days storage (data not shown). The mean DM increased during storage, being significant solely in non UV-B-treated roots (11%) (Fig. 6a). In turn, TSS substantially accumulated in untreated and UV-B treated roots, reaching the highest values of 18.2 °Brix in treated roots of ‘Monty Rz’ upon 7 days of storage. This represents increases of 11.4 and 12.9% in ‘Monty Rz’ and ‘Belushi Rz’, respectively, compared with the values of untreated roots at time 0 (Fig. 6b).

## Discussion

### Elicited betanin accumulation upon ethephon treatment

In the first part of this study, we demonstrated that foliar application of ethephon elicited betanin accumulation in the roots of the two *Beta vulgaris* L. ssp. *vulgaris* cultivars Monty Rz and Belushi Rz (Table 3; Fig. 3a and b). In previous years, in vitro elicitation of betalain in red beet hairy root cultures has been achieved [25–28]. However, to the best of our knowledge, there are no articles reporting enhanced betalain accumulation following elicitation in studies of red beet plants in vivo. A correlation between foliar application of ethephon and enhanced anthocyanin accumulation in roots has recently been reported in black carrot [19]. Therefore, the present study supports the existence of common regulatory networks for anthocyanin and betalain synthesis, and reinforces the application of ethephon and similar ethylene-generating compounds for natural food colourant elicitation.

In beetroot, betanin is a major antioxidant and acts as a strong scavenger of ROS [29–31]. In this respect, ethylene-induced ROS accumulation has been extensively reported in different plant species [32, 33]. Thus, increased betanin content can be a protective response to excess of ROS following ethephon treatment. The fact that vulgaxanthin content did not increase in response to ethephon can be linked to its moderate radical-scavenging activity compared to betacyanins [29, 31], due to the lack of phenolic hydroxyl groups in vulgaxanthin structure [34].

### Accumulation of betalains and phenolic compounds during beetroot growth

The present results documented differentiated accumulation kinetics of Bn and Vx during root growth. Bn content displayed a peak at 6 weeks after sowing, followed by a continuous decrease until the end of the growing period. In contrast, vulgaxanthin content increased constantly. These kinetics are consistent with those previously reported for beetroot of different cultivars [4, 8, 35]. As a consequence, higher Bn:Vx were achieved at early stages of growth (38 to 46), at 6 weeks after sowing, which progressively decreased to values between 3.8 and 4.9 at the end of the growing period (Fig. 3e and f). Herein, ethephon application increased Bn:Vx in both cultivars. Together with total Bn and Vx contents, Bn:Vx determine the colour hue of beetroot extract. Since higher ratios are reported as more suitable for colourant production [8], increased Bn:Vx upon ethephon treatment enhances the profitability of beetroot extract.

Besides betalains, other relevant phenolic compounds reported in red beet are gallic, syringic, caffeic acids and flavonoids [13, 15]. In the present study, increased TPC was reported upon ethephon treatment (Table 3; Fig. 3g and h). In general, the monitoring of TPC and Bn content showed similarities during root growth. The most outstanding difference between TPC and betalain accumulation curves occurred between 3 and 6 weeks after sowing, when TPC remained unvariable (Fig. 3g and h) and Bn content increased substantially (Fig. 3a and b). This may indicate that, at early stages of growth, non-betalainic phenolic compounds have a higher preponderance than in later stages of growth.

### Physiological significance of sugar contents during betalain accumulation

Betalain pigments underlie glycosylation of cyclo-DOPA and betalamic acid, in which sugar molecules are added. In our work, the application of ethephon increased betanin concentration and significantly decreased TSS of beetroots (Fig. 3), which may result from increased sugar consumption for betanin biosynthesis. Moreover, various studies have pointed out the role of sugars as signalling molecules in the biosynthesis of phenolic compounds [36–38]. Likewise, a peak in sugar concentration has been reported to be concomitant with increased anthocyanin content in black carrot [19] and Arabidopsis [39, 40]. Our results support these observations, since the highest TSS accumulation rate in untreated and treated plants of both beetroot cultivars occurred simultaneously with the fastest phase of Bn accumulation (from 3 to 6 weeks after sowing).

Nevertheless, further studies involving whole red beet plants are needed to understand the kinetics of sugar metabolism in shoots and roots.

### Correlation between expression of betalain biosynthesis-related genes and betalain accumulation

To our knowledge, the overexpression of biosynthesis-related betalain genes upon elicitation has not been found previously. In the light of the expression studies on ethephon treated beetroots of ´Monty Rz’ and ‘Belushi Rz’ 16 weeks after sowing (Fig. 4), we hypothesise that released ethylene acts as an inducer of the betalain biosynthetic pathway through the activation of *Bv*MYB1. These results corroborate recent findings in black carrot, where the expression of the *BvMYB1* homologous, *DcMYB1*, and the anthocyanin biosynthesis genes were induced following ethephon elicitation [19], and support the existence of common regulatory mechanisms in betalains and anthocyanins biosynthesis [41].

Remarkably, DODA1, which lead to the formation of betalamic acid, the basic backbone of red and yellow betalain biosynthesis, [16, 42], reached identical levels (5.2-fold higher transcript levels in treated compared to untreated plants) in both cultivars (Fig. 4). In contrast, the expression levels of *BvCYP76AD1* and *BvCYP76AD6* differed between cultivars. In ‘Monty Rz’ *BvCYP76AD1* and *BvCYP76AD6* showed 4.9 and 5.3-fold higher transcripts, respectively, than in untreated plants (Fig. 4a), while those levels in ‘Belushi Rz’ were comparable between untreated and ethephon-treated plants (Fig. 4b). As the cytochrome P450 enzymes (*Bv*CYP76AD1, *Bv*CYP76AD5 and *Bv*CYP76AD6) redundantly catalyse the hydroxylation of tyrosine to form L-DOPA (the initial step in betalain biosynthesis) [16, 17], the different expression patterns of these enzymes between cultivars may reflect distinct regulatory mechanisms.

### Increased Bn:Vx and TPC during short-time beetroot storage

In the second part of this study, we showed a decrease of Vx content upon short-time storage at 15 °C and 98–100% RH, whereas Bn content remained unchanged (Fig. 5a and b). Thus, increased Bn:Vx were achieved (Fig. 5c and d), which in turn may improve the profitability of beetroot for colourant production [8]. To our knowledge, no previous work has reported decreased betaxanthin levels while betacyanin contents remained constant in the postharvest environment. Although temperatures between 2 and 4 °C are generally recommended for long-time storage of beets, higher temperatures may be favouring the degradation kinetics of Vx under our experimental conditions.

In addition, exposure of beetroots to a UV-B radiation fluence of 1.23 kJ m^− 2^ induced significant increases up to 15.5% of TPC in ‘Belushi Rz’ (Fig. 5f). Similar UV-B radiation fluences (1.304 kJ m^− 2^) were reported to enhance phenolic content of sliced carrots [43]. Using this low UV-B fluence, the exposure time to UV-B radiation is much shorter, which minimizes the heating of roots and avoids water loss. Phenolic compounds have photo-protective roles because of their UV-absorbing properties and their ability to act as antioxidants [44]. Since UV-B does not efficiently penetrate into deeper tissues, the outer beetroot cell layers (peel and crown) are the tissues absorbing and potentially responding to UV-B radiation and increasing TPC. Further studies involving separated root tissue samples will allow a better characterisation of the response to UV-B radiation.

In summary, our findings demonstrate that field application of ethephon on ‘Monty Rz’ and ‘Belushi Rz’ beetroots results in increased betanin per unit of biomass and betanin to vulgaxanthin ratio, and decreased TSS. Furthermore, the patterns of expression of betalain biosynthetic genes and the *BvMYB1* transcription factor correlated with that of betalain accumulation. These facts reinforce the existence of common regulatory networks for anthocyanin and betalain synthesis. In the postharvest environment, a low UV-B fluence treatment of the roots, followed by short-time storages for 3 and 7 days resulted in increased TPC and Bn:Vx, without detrimental effects on beetroot quality.

## Conclusions

Field-applied ethephon and postharvest UV-B radiation improved quality of beetroot by increasing betanin to vulgaxanthin ratio, betanin and phenolic contents, and decreasing soluble solids content. Betanin content in ethephon-treated beetroots correlated to increased expression of betalain biosynthetic genes and the betalain pathway activator *BvMYB1*.

## Abbreviations

Bn: Betanin
Bn:Vx: Betanin to vulgaxanthin ratio
DM: Dry matter
PCR: Polymerase chain reaction
RH: Relative humidity
TPC: Total phenolic content
TSS: Total soluble solids content
UV-B: Ultraviolet B
Vx: Vulgaxanthin I
